# Movement-Evoked Pain and Temporal Summation in Individuals with Symptomatic Rotator Cuff Tears: A Cross-Sectional Study

**DOI:** 10.3390/life15091394

**Published:** 2025-09-03

**Authors:** Anupama Prabhu B, Arun G. Maiya, Vivek Pandey, Kiran K. V. Acharya, Vennila Jaganathan, James M. Elliott, Mira Meeus

**Affiliations:** 1 Department of Physiotherapy, Manipal College of Health Professions, Manipal Academy of Higher Education, Manipal 576104, India; anupama.prabhu@manipal.edu; 2 Department of Orthopaedics, Kasturba Medical College, Manipal Academy of Higher Education, Manipal 576104, India; vivek.pandey@manipal.edu (V.P.); kiran.acharya@manipal.edu (K.K.V.A.); 3 Department of Statistics, Manipal College of Health Professions, Manipal Academy of Higher Education, Manipal 576104, India; vennila.j@manipal.edu; 4 The Kolling Institute, Northern Sydney (Arabanoo) Precinct, St Leonards, NSW 2065, Australia; james.elliott@sydney.edu.au; 5 Sydney School of Health Sciences, The University of Sydney, Camperdown, NSW 2050, Australia; 6 MOVANT Research Group, Department of Rehabilitation Sciences and Physiotherapy (REVAKI), University of Antwerp, 2610 Antwerp, Belgium; mira.meeus@uantwerpen.be; 7 Pain in Motion, University of Antwerp, 2610 Antwerp, Belgium

**Keywords:** pain, quantitative sensory testing, shoulder, tendon

## Abstract

Musculoskeletal shoulder pain due to rotator cuff (RC) tears is a prevalent condition that significantly impacts function and quality of life. Understanding the underlying pain mechanisms, including movement-evoked pain (MEP) and pain facilitation phenomena such as temporal summation (TS), is essential for improving targeted interventions. This cross-sectional study examined relationships among TS, pain at rest, and MEP in 85 individuals with symptomatic RC tears. Mechanical TS was assessed on the contralateral forearm using standardized mechanical stimuli, while pain at rest and MEP during active arm elevation were measured via numerical rating scales. Spearman’s correlations were performed for the overall cohort and stratified by pain duration (<3 months, acute; ≥3 months, chronic). Weak but statistically significant correlations were found between TS and MEP (r = 0.23, *p* = 0.02) and between pain at rest and MEP (r = 0.30, *p* = 0.005), whereas no correlation existed between TS and pain at rest. The logistic regression model showed limited predictive ability. These exploratory findings suggest partially overlapping but distinct pain mechanisms in RC tear patients and should be interpreted as hypothesis-generating, warranting validation in larger, prospective cohorts.

## 1. Introduction

Rotator cuff (RC) tears and their related symptoms impose a significant burden on the healthcare system, causing 28.8% of individuals to seek consultation with general practitioners for shoulder pain [1]. RC tears are common contributors to shoulder discomfort and impairment and become more prevalent as individuals age [2]. Despite extensive research, the exact cause of shoulder pain resulting from RC tears remains unclear. Asymptomatic RC tears were found to be twice as prevalent as symptomatic RC tears in the general population [3]. These findings add to the complexity of understanding shoulder pain associated with RC tears. The relationships among the severity of RC tears, pain, and disability experienced by individuals are complex and vary from person to person. Hence, findings on imaging modalities do not align with patient-reported pain measures.

Recent research has reported that the prevalence of central sensitization in individuals with chronic RC tears is 39.4%, which is higher than that reported in individuals with spine and knee musculoskeletal disorders [4]. The reported prevalence of central sensitization syndrome in RC tear patients was established using the central sensitization inventory, with a cutoff score of ≥40 indicating central sensitization syndrome positivity. This cross-sectional study employed the central sensitization inventory to capture symptom severity related to central sensitization, enabling standardised patient classification and identifying associations with clinical factors such as symptom chronicity and psychological comorbidities [4]. However, the evidence concerning central sensitization in rotator cuff-related shoulder pain (RCRSP) remains ambiguous [5], likely because of the inherent difficulties in accurately and consistently measuring central sensitization directly in humans. Compared with those at rest, individuals with RCRSP often experience increased pain levels when moving their shoulders [6]. Recent studies have highlighted the importance of measuring movement-evoked pain (MEP) in musculoskeletal disorders [7]. MEP represents a significant yet commonly ignored obstacle to patient adherence to exercise programs [8]. The mechanisms underlying pain at rest and MEP are also distinct [9]. Peripheral and central sensitization processes seem to contribute to the development of MEP [10,11]. The term “sensitivity to MEP” refers to the heightened experience of pain during a movement in reaction to repeated movements, such as repeatedly raising the arm [12]. However, research has revealed no correlation between the number of abnormal RC tendons and the occurrence of pain triggered by movement in the shoulder [13,14].

Temporal summation (TS) refers to the neurophysiological process whereby repetitive noxious stimuli delivered in rapid succession leads to an increased excitability of dorsal horn neurons in the spinal cord. This occurs through the summation of excitatory postsynaptic potentials at a single synapse, resulting in progressively enhanced depolarisation that can reach the threshold to elicit action potentials. This phenomenon, often termed “wind-up”, reflects central facilitation of pain processing, primarily mediated by C-fibre nociceptive afferents and involves mechanisms such as N-methyl-D-aspartate receptor activation and calcium influx [15]. It is a key experimental indicator of central sensitization and increased central nervous system responsiveness to pain. In recent years, studies have begun to directly investigate central sensitization in RCRSP populations, although reported prevalence rates vary widely depending on assessment tools and patient samples. For instance, current evidence suggests that features of central sensitization—such as elevated TS and reduced conditioned pain modulation—may be present in both persistent and acute shoulder pain cohorts, but further research is needed to clarify these relationships and their relevance to clinical pain outcomes. Importantly, a growing body of shoulder-specific research now supports the plausibility of a mechanistic link between pronounced TS and heightened MEP, particularly in patients experiencing persistent symptoms, even as the strength of this relationship remains a topic of ongoing investigation. Evidence suggests that MEP reflects, in part, facilitatory pain processing within the central nervous system. Prospective studies show that enhanced TS of mechanical pain, a model of central facilitation and spinal wind-up, predicts greater MEP severity in individuals with chronic musculoskeletal disorders, even after accounting for resting pain symptoms [16]. Thus, while TS and MEP are distinct phenomena, emergent data support TS as an indicator of the central mechanisms that partly underlie MEP in musculoskeletal disorders.

In many chronic pain conditions, including RCRSP, everyday movements repeatedly activate peripheral nociceptors in a way that mimics the brief, repeated pulses used in TS paradigms. These patients often exhibit exuberant TS (i.e., greater increases in pain ratings across the pulse train) because their spinal circuits are primed. Spinal circuits become “primed” in chronic pain patients through convergence of synaptic plasticity, loss of inhibitory control, neuroimmune activation, and altered descending modulation. These changes lower the threshold for dorsal horn neurons to fire and to summate successive C fibre inputs so that even normal or mildly noxious afferent volleys produce exaggerated pain. As such, these patients may be prone to amplified pain during repetitive arm movements. By identifying individuals with pronounced TS, clinicians can both anticipate which patients will struggle most with activity-related pain and tailor interventions, such as graded exposure, to target the underlying central facilitation. In RCRSP, however, it remains unclear whether the severity of MEP is significantly related to TS. Therefore, this study aims to establish the relationships among TS, pain at rest, and MEP in individuals with RC tears.

## 2. Materials and Methods

### 2.1. Study Design

This cross-sectional study was conducted within a prospective longitudinal study that aimed to develop a prediction model for the development of chronic pain in individuals undergoing arthroscopic rotator cuff repair (ARCR). Data collection was performed preoperatively before the ARCR procedure. This study was conducted between August 2022 and January 2024. Institutional ethics committee (Approval number IEC 40/2020) approval was obtained before the commencement of the study. The trial was registered under the clinical trial registry with a registration number (CTRI/2021/04/032929). All participants received a participant information sheet and signed a written informed consent form before participating in the study. The study was conducted per the Helsinki Declaration.

### 2.2. Study Population

The study inclusion criteria were individuals aged 18 to 70 years, with unilateral symptomatic RC tears diagnosed through clinical examination and/or diagnostic imaging (diagnostic ultrasound/magnetic resonance imaging), and participants with RC tears resulting from degenerative and traumatic causes. The exclusion criteria included a history of shoulder surgery; other shoulder disorders, such as osteoarthritis, instability, labral tear, or infection; a diagnosis of psychological disorders; chronic pain in other body regions; systemic inflammatory conditions; neurological disorders; or malignancy.

### 2.3. Procedure

Demographic characteristics, including age, sex, height, weight, duration of symptoms, affected side, and dominant side, were recorded for all participants who agreed to participate in the study. The participants were given a standardized briefing to introduce them to the numerical pain rating scale (NRS). A familiarization session with the TS was conducted before data collection, which included a trial of the TS using a mechanical stimulus on the contralateral knee, specifically targeting the tibialis anterior muscle. All the data were obtained by a qualified physiotherapist (APB) with 12 years of clinical experience.

### 2.4. Temporal Summation

The participants were told to use an NRS from 0 to 10, with zero indicating “no pain” and 10 denoting “most intense imaginable pain”. Each participant was subsequently instructed to rate their level of pain at the test location on a 0–10 scale. Mechanical TS was assessed on the dorsal aspect of the unaffected forearm. The TS was assessed mechanically at a remote site to evaluate generalized central pain facilitation, avoiding confounding effects of local tissue pathology at the shoulder. The mechanical stimuli were delivered via a Semmes Weinstein monofilament log 6.65, which was calibrated to bend at 300 g of pressure. The monofilament was used to deliver stimuli, and it was positioned vertically above the target site of contact. Initially, the researcher applied a single pinprick stimulus, followed by a series of 10 pinprick stimuli delivered at a rate of 1 stimulus per second within a 1 cm^2^ area using a metronome. Pain ratings were recorded after the first and tenth stimuli. This procedure was repeated three times, and the average TS difference (TS_D_) score was used for analysis. The TS_D_ score was calculated by subtracting the first stimulus’s pain intensity rating (TS_1_) from the tenth stimulus’s intensity rating (TS_10_) [17]. The reproducibility of TS testing may be influenced by several methodological factors, including the type and intensity of the stimulus, the anatomical site of assessment, participant characteristics (e.g., anxiety, attention), and interrater or intra-rater variability. Although the TS is widely used in pain research, studies indicate that its test–retest reliability can range from moderate to good, but it is not absolute, especially in clinical populations with musculoskeletal pain. We acknowledge limitations related to test–retest variability. It is notable that the Semmes Weinstein monofilaments employed, specifically the 300 g (log 6.65) filament, have demonstrated high test–retest reliability in previous studies, with intraclass correlation coefficients (ICC) ranging from 0.84 to 0.93 [18,19]. TS assessments similarly show good to excellent reliability, with reported ICCs between 0.75 and 0.97 depending on the stimulus and measurement protocols [20]. In this study, we sought to minimize variability by standardizing the testing procedure, training a single assessor, and conducting a familiarization session for all participants.

### 2.5. Movement-Evoked Pain

The participants provided their pain ratings on the NRS for current pain at rest and MEP. Pain at rest was assessed while the patient sat upright on the edge of the bed without using any support or sling for the affected shoulder. Measuring pain at rest as current pain intensity while seated immediately before performing a task as baseline pain is suggested, as it allows for direct comparison between pain at rest and MEP [21]. MEP was assessed during active arm elevation performed in the scapular plane (scaption). This plane was anatomically standardized by instructing participants to elevate their arm approximately 30 to 45 degrees anterior to the frontal plane, with the thumb pointing upward, representing the functional scapular plane. The plane and movement were demonstrated and explained to participants to ensure consistent performance. This standardization aligns with clinical and biomechanical definitions of scaption, facilitating reproducible and relevant shoulder movement assessment. The pain intensity was recorded at the end of the available movement. This trial was repeated three times on the affected shoulder, with a rest period of two minutes between each repetition. The average intensity of the three trials was used to calculate MEP. Higher values represented greater pain ratings on the NRS. Testing MEP while the shoulder is in active motion until the onset of pain or at a maximum range of motion has been established as a reliable method for evaluating MEP in patients with RCRSP [22].

### 2.6. Statistical Analysis

Jamovi 2.3.26 software was used for the statistical analysis. Descriptive statistics were employed to present participant demographic data. As the data did not follow a normal distribution, nonparametric statistics were used according to the Kolmogorov–Smirnov test. The Mann–Whitney U test was used to investigate the differences in pain at rest, MEP, and TS between people with acute and chronic pain. The relationships between TS, movement-evoked pain, and pain at rest in RC tear participants were evaluated via Spearman’s rank correlation coefficient. The values were expressed as correlation coefficients and interpreted as described by Hinkle et al. (2003), where ‘0’ means no relationship, ‘0.00 to 0.30’ means little if any correlation, ‘0.30 to 0.50’ means a low positive correlation, ‘0.50 to 0.70’ means a moderate positive correlation, ‘0.70 to 0.90’ means a high positive correlation, and ‘0.90 to 1.00’ means a very high positive correlation [23]. Logistic regression analysis was performed to determine the associations between TS, MEP, and pain at rest in participants subgrouped into acute pain (<3 months) and chronic pain (≥3 months). The level of significance was set at *p* ≤ 0.05.

## 3. Results

This cross-sectional study examined 85 participants, and Table 1 displays the characteristics of the participants. Most of the participants were middle-aged males who were right-hand dominant, and nearly half of them experienced pain on the dominant side. The duration of symptoms varied widely, and participants with both acute (*n* = 40) and chronic (*n* = 45) pain were included. Pain duration was categorized using a 3-month threshold to distinguish acute (<3 months) from chronic (≥3 months) pain. This cutoff is widely accepted in clinical pain research and reflects the conventional boundary at which pain is considered chronic, as recommended by the International Association for the Study of Pain and commonly utilized in musculoskeletal pain studies. The average pain score indicates greater pain intensity during movement than at rest. The mean scores of TS at the end of the first and tenth stimuli are displayed alongside the mean difference score for TS (TS_D_ = TS_10_ − TS_1_).

Table 2 displays the correlations between TS, pain at rest, and MEP in participants with RC tears. A weak, statistically significant correlation was found between the mechanical TS and MEP (r = 0.23; *p* = 0.02). MEP and pain at rest also demonstrated a weak, statistically significant correlation (r = 0.30; *p* = 0.005), whereas TS and pain at rest were not correlated (r = 0.04; *p* = 0.68). Although the correlations between TS and MEP and between pain at rest and MEP reached statistical significance, their weak magnitude indicates only modest associations. These results suggest partially overlapping but distinct pain mechanisms in RC patients.

The Mann–Whitney U test was performed to examine the differences in pain at rest, MEP, and TS between individuals with acute and chronic pain; the results are shown in Table 3. There were no statistically significant differences between chronic and acute groups for pain at rest (*p* = 0.560), MEP (*p* = 0.075), or TS (*p* = 0.612). However, MEP showed a small to moderate effect size (0.22), suggesting a trend toward higher MEP in the chronic group despite the non-significant *p*-value. Pain at rest and TS had negligible effect sizes, indicating minimal differences between groups.

As shown in Table 4, the logistic regression analysis revealed various associations between different pain durations and factors. A relatively high odds ratio was observed for pain at rest (1.018 with CI (0.82–1.264)) and TS (1.153 with CI (0.901–1.475)), suggesting a potential association between pain at rest, TS, and the presence of acute or chronic pain. In contrast, MEP had a lower odds ratio (0.773 with CI (0.599–0.998)), indicating an inverse relationship with acute and chronic pain.

Despite these associations, the analysis also revealed that, within the subgroup of participants with acute and chronic pain, there was no significant association between TS and pain at rest. The model demonstrated mild predictive power, with an area under the curve (AUC) of 0.600 and a nonsignificant *p*-value (0.189), suggesting limited predictive ability. Furthermore, the model exhibited a low McFadden’s R2 value (R2McF = 0.04), indicating that it explained only a small proportion of the variance in pain outcomes. This poor performance suggests that additional variables beyond TS, pain at rest, and MEP are likely needed to adequately predict pain chronicity in this population.

**Figure 1:**
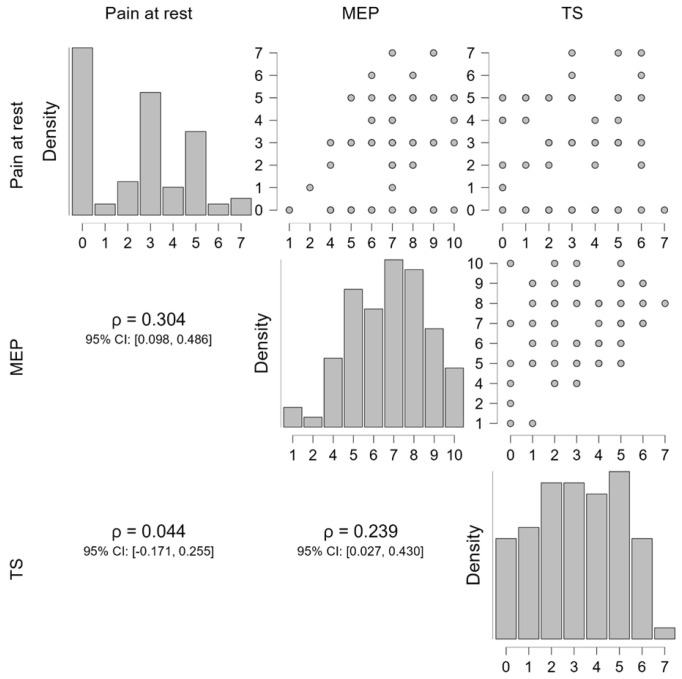
Scatter plots, confidence intervals, and correlation maps of the correlations between pain at rest, MEP, and TS.

**Figure 2:**
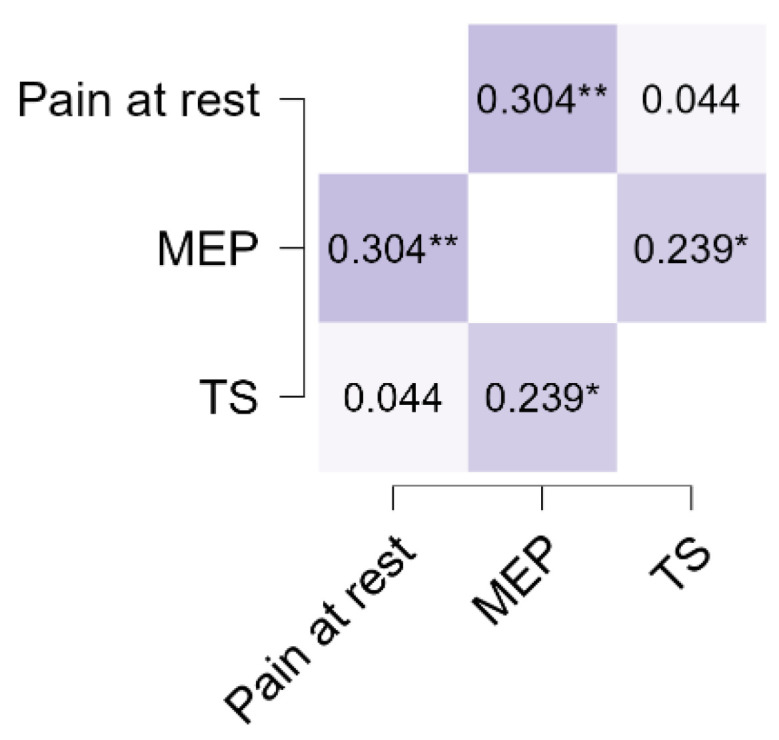
Spearman’s rho heatmap demonstrating the correlation between pain at rest, MEP, and TS. * *p* < 0.05, ** *p* < 0.01.

Figure 1 and Figure 2 illustrate the relationships between pain at rest, MEP, and TS. Spearman’s rho correlations showed a moderate positive correlation between MEP and TS, indicating that individuals with higher MEP tended to report greater TS. In contrast, pain at rest demonstrated weak correlations with both MEP and TS. These findings suggest that dynamic pain measures such as MEP and TS may be more closely related than static pain measures like pain at rest. Although some associations are statistically significant, their effect sizes are small, suggesting limited clinical relevance. These findings highlight modest relationships that warrant cautious interpretation within the complex framework of pain mechanisms and patient variability.

## 4. Discussion

This study investigated the correlations among TS, pain at rest, and MEP in individuals with symptomatic RC tears. The study revealed only weak but statistically significant correlations between TS and MEP and between pain at rest and MEP. These weak associations, which are consistent with those reported in previous pain mechanism studies, highlight the complex and multifaceted nature of pain in patients with RC tears. As such, the clinical implications of these findings are limited and should be interpreted with caution. These results are hypothesis-generating and warrant confirmation in larger, focused studies.

While TS is a useful indicator of central pain facilitation (wind-up), it does not capture all aspects of central sensitization, such as altered descending inhibitory control or neuroimmune modulation. Central sensitization encompasses a complex interplay of neurophysiological mechanisms that contribute to amplified pain processing within the central nervous system. These mechanisms include increased excitability of spinal dorsal horn neurons through wind-up and TS, dysregulation of descending inhibitory pathways, activation of glial cells and neuroimmune signalling, and nociplastic changes spanning the spinal cord and brain. TS captures the phenomenon of spinal wind-up but does not fully represent the multifactorial nature of central sensitization. Additionally, our choice to assess TS on the contralateral forearm was intended to evaluate generalized facilitation, but it precludes direct inference about segmental or localized sensitization at the shoulder. Future studies should combine local and remote quantitative sensory testing measures for a more comprehensive assessment. According to a recent study comparing quantitative sensory testing measurements with MEPs in persistent low back pain patients, TS explained 12% of the variation in MEP [24]. In whiplash injuries, TS could account for 20% of the variance in sensitivity to MEP, suggesting that certain processes underlying MEP also intersect with TS processes [12]. In individuals with knee osteoarthritis, the association between sensitivity to physical activity and TS on the index knee was relatively modest [25]. The pain-movement conceptual model emphasizes the interdependence between pain and movement [26]. A recent study revealed that an imbalance in pain modulation, characterized by increased pain facilitation detected through TS, could significantly contribute to the severity of movement-related pain and impaired physical function in individuals with chronic low back pain [16]. A significant association was found between the TS of mechanical pain and the severity of clinical pain in adults suffering from chronic low back pain [27]. A deeper understanding of how facilitatory pain pathways influence MEP can lead to improvements in clinical practice and research. Even though the intensity of the mechanical stimuli remained constant throughout the TS procedure, RC tear patients reported noticeably more pain in response to the last application of the stimulus than the first. The weak linear correlation between the TS and MEP in our study points towards two distinct mechanisms for pain during movement and the facilitating process of ascending pain. The modest strength of associations highlights the multifactorial nature of pain in RC tear patients and underscores the need for comprehensive assessment approaches to better delineate underlying mechanisms. Importantly, TS measurements are subject to inherent variability. Factors such as subtle differences in stimulus application, individual participant response variability, and contextual influences (e.g., mood, prior pain experience) can all affect TS outcomes and may partially account for the modest correlations observed in this study. Nevertheless, despite these limitations, the TS remains a useful experimental tool for investigating central pain facilitation processes.

It has been suggested that changes in the central nervous system, lowering nociceptive thresholds, and peripheral mechanical factors, such as the activation of silent nociceptors, are responsible for MEP [11,28]. Unlike self-reported validated questionnaires, the MEP provides a pain rating immediately after completing a physical activity. The observed weak, statistically significant correlation between MEP and pain at rest suggests that these two pain experiences, although related, are distinct and likely influenced by different underlying mechanisms. A study examining the association between pain at rest and MEP in knee osteoarthritis patients concluded that there was an absence of correlation between these two pain phenomena [21]. Pain at rest is frequently associated with ongoing inflammation or tissue damage, which leads to persistent nociceptor activation even in the absence of movement. In contrast, MEP involves the activation of previously silent nociceptors that specifically respond to mechanical stimuli during movement [28]. This activation can occur even without overt inflammation, indicating a distinct peripheral sensitization process. Furthermore, MEP has been linked to central sensitization, where heightened sensitivity can amplify pain perception during movement, also affecting pain at rest. Recent evidence highlights the role of central sensitization as a contributing factor to persistent pain in shoulder disorders. In a study of patients undergoing shoulder surgery, central sensitization was significantly more prevalent among individuals with glenohumeral osteoarthritis, and female sex, pain intensity, and symptom duration emerged as independent risk factors, reinforcing the need to consider central mechanisms in chronic shoulder pain assessment and management [29]. Given the exploratory nature of this secondary analysis and the modest predictive and statistical association observed, these results are best considered hypothesis-generating. They provide preliminary direction for future dedicated studies but should not be interpreted as having immediate clinical applicability without independent replication. In summary, the weak correlation between MEP and pain at rest reflects their distinct underlying peripheral pathophysiological mechanisms. Recognizing and assessing both types of pain can inform more comprehensive pain management strategies.

The present findings suggest differential associations between pain characteristics and acute and chronic pain states. Pain at rest and TS were positively associated with the presence of acute or chronic pain, although these associations did not reach statistical significance. These findings are partially consistent with those of previous studies, indicating that central sensitization mechanisms, of which TS is a hallmark, are often elevated in individuals with chronic musculoskeletal pain [30]. MEP, in contrast, showed a statistically significant inverse association, which may reflect the protective behavior observed in chronic pain states where individuals avoid movement to reduce perceived threat or discomfort [31]. The observed inverse association for MEP may reflect complex neurophysiological and behavioural adaptations. Individuals experiencing higher resting pain may adopt protective movement strategies that limit pain provocation during arm elevation. Additionally, central and peripheral mechanisms of pain modulation are multifaceted, involving both facilitatory and inhibitory influences that can produce counterintuitive relationships. Psychological factors, such as fear-avoidance or altered pain perception, may further mediate these findings. Measurement variability and sample characteristics could also contribute to this result. Thus, inverse association underscores the complexity of pain movement interactions in RC tear patients and highlights the need for multifactorial assessment approaches.

### 4.1. Study Limitations

This study has several important limitations that must be considered when interpreting its findings. First, it represents a secondary analysis of baseline data from a longitudinal cohort originally designed for other primary aims and thus lacks a dedicated sample size calculation specific to the correlations of interest; this may have reduced the statistical power and validity of associational findings. Consequently, some variables of interest may not have been collected for the current analysis, and the dataset may lack certain covariates that could influence the outcomes. As a result, all correlation and regression analyses should be considered exploratory. The assessment of TS was limited to a mechanical stimulus applied to the contralateral forearm, potentially capturing only generalized central sensitization and not localized segmental processes relevant to the affected shoulder. Moreover, the use of a Semmes–Weinstein monofilament, rather than a standardized pin-prick device, may have influenced the sensitivity of TS detection. Future studies should incorporate a multimodal quantitative sensory testing battery assessing TS and other sensory phenomena both locally and remotely to differentiate between peripheral and central mechanisms. MEP was assessed solely in the scaption plane and may not reflect pain responses during other shoulder movements. Future studies can incorporate multidirectional movement assessments to capture a more comprehensive MEP profile. There was also considerable heterogeneity in symptom duration among participants, and although subgroup analyses were undertaken, the sample size within acute and chronic subgroups limits the interpretability of these results. Pain medication use was recorded but not standardized or controlled, introducing potential confounding factors. Finally, all participants were right-hand dominant, which may affect generalizability, and the modest predictive power of regression models further limits the immediate clinical applicability of these findings. Hand dominance can influence shoulder kinematics, neuromuscular control, and possible pain processing patterns; therefore, our results should be interpreted with caution when extrapolating to populations with left dominant profiles. Collectively, these methodological constraints indicate that the present results should be interpreted as preliminary, and further research with larger, more homogeneous, and prospectively designed samples is warranted.

### 4.2. Implications

Although this study provides preliminary insights into the interplay between TS, MEP, and pain at rest in individuals with symptomatic RC tears, the consistently weak associations and poor predictive performance of regression models suggest that these measures should not yet be relied upon as stand-alone clinical indicators of central sensitization or pain chronicity in this population. The findings highlight the multifactorial and complex nature of RC tears, where overlapping but distinct mechanisms are likely to contribute to the patient’s experience. As such, clinicians should continue to employ comprehensive, multidimensional assessments—incorporating patient history, functional testing, and broader psychosocial factors—when evaluating and managing RCRSP. Until future research clarifies the utility and reliability of TS and MEP as clinical tools, their use should be interpreted with caution and viewed as one component within an individualized, patient-centered approach to care.

## 5. Conclusions

This study revealed a weak but significant correlation between MEP and TS in individuals with symptomatic RC tears, suggesting partially overlapping yet distinct underlying pain mechanisms. No association was observed between pain at rest and TS, supporting the hypothesis that different neurophysiological pathways may drive resting and MEP. However, owing to the modest strength of these associations and the exploratory nature of this analysis, the findings should be interpreted cautiously, and further research is needed to confirm their clinical relevance.

## Figures and Tables

**Table 1:** General characteristics of the participants (*n* = 85).

Characteristics	Median (IQR)
Age (Years)	55 (9)
Height (cm)	161 (14)
Weight (kg)	70 (13)
Pain at rest	3 (4)
MEP	7 (3)
TS_1_	1 (1)
TS_10_	5 (3)
TS_D_	3 (3)
Duration of symptoms (Months)	3 (5)
Gender (Male/Female)	54 (63.5%)/31 (36.5%)
Affected side (R/L)	46 (54.1%)/39 (45.9%)
Dominant side (R/L)	85 (100%)/0 (0%)

**Table 2:** Spearman’s (r) correlation between TS, pain at rest, and MEP.

Variable		Pain at Rest		MEP		TS
Pain at rest	Spearman’s rho					
	*p* value					
	Upper 95% CI					
	Lower 95% CI	—				
	Effect size (Fisher’s z)	—				
	SE Effect size	—				
MEP	Spearman’s rho	0.304	**	—		
	*p* value	0.005		—		
	Upper 95% CI	0.486		—		
	Lower 95% CI	0.098		—		
	Effect size (Fisher’s z)	0.314		—		
	SE Effect size	0.112		—		
TS	Spearman’s rho	0.044		0.239	*	—
	*p* value	0.688		0.028		—
	Upper 95% CI	0.255		0.430		—
	Lower 95% CI	−0.171		0.027		—
	Effect size (Fisher’s z)	0.044		0.244		—
	SE Effect size	0.110		0.112		—

* *p* < 0.05, ** *p* < 0.01.

**Table 3:** Differences between pain at rest, MEP, and TS in individuals with acute and chronic pain.

	Group	*N*	Mean	SD	Statistic	*p*	95% Confidence Interval		Effect Size
Pain at rest	Chronic	45	2.56	2.14	836	0.560	−0.00000388	1.000	0.0717
	Acute	40	2.33	2.19					
MEP	Chronic	45	7.09	1.92	700	0.075	−0.00000129	2.000	0.2228
	Acute	40	6.30	2.00					
TS	Chronic	45	3.04	1.99	843	0.612	−1.00	1.000	0.0639
	Acute	40	3.27	1.81					

**Table 4:** Association between pain at rest, TS, and MEP among the subgroup of individuals with acute and chronic pain.

Predictor	Estimate	SE	Z	*p*	Odds Ratio	95% Confidence Interval	
						Lower	Upper
Pain at rest	0.018	0.111	0.163	0.871	1.018	0.82	1.264
MEP	−0.2571	0.13	−1.978	0.048	0.773	0.599	0.998
TS	0.1421	0.126	1.132	0.258	1.153	0.901	1.475

Note: Estimates represent the log odds of “Category = 1” vs. “Category = 0”. Category = 1: acute pain; Category = 0: chronic pain; SE = standard error; Z = test statistic value from the logistic regression analysis; *p* = *p*-value indicating statistical significance.

## Data Availability

The data presented in this study are available upon request from the corresponding author. The data are not publicly available for privacy reasons.

## Abbreviations

The following abbreviations are used in this manuscript:

MEP	Movement-Evoked Pain
RC	Rotator Cuff
TS	Temporal Summation
RCRSP	Rotator Cuff Related Shoulder Pain
ARCR	Arthroscopic Rotator Cuff Repair
NRS	Numerical Pain Rating Scale
